# Intergroup Biases in Fear-induced Aggression

**DOI:** 10.3389/fpsyg.2017.00049

**Published:** 2017-01-24

**Authors:** Nobuhiro Mifune, Dora Simunovic, Toshio Yamagishi

**Affiliations:** ^1^School of Economics and Management, Kochi University of TechnologyKochi, Japan; ^2^Bremen International Graduate School of Social SciencesBremen, Germany; ^3^Graduate School of International Corporate Strategy, Hitotsubashi UniversityTokyo, Japan

**Keywords:** preemptive strike, intergroup aggression, outgroup derogation, minimal groups, ingroup favoritism

## Abstract

Using a recently created preemptive strike game (PSG) with 176 participants, we investigated if the motivations of spite and/or fear promotes aggression that requires a small cost to the aggressor and imposes a larger cost on the opponent, and confirmed the earlier finding that fear does but spite does not promote intergroup aggression when the groups are characterized as minimal groups; additionally, the rate of intergroup aggression did not vary according to the group membership of the opponent. The PSG represents a situation in which both the motivations of spite and of fear can logically drive players to choose an option of aggression against an opponent. Participants decide whether or not to attack another participant, who also has the same capability. The decision is made in real time, using a computer. We discuss theoretical implications of our findings on the evolutionary foundations of intragroup cooperation and intergroup aggression. The evolutionary model of intergroup aggression, or the parochial altruism model, posits that intragroup cooperation and intergroup aggression have co-evolved, and thus it predicts both intragroup cooperation and intergroup aggression to emerge even in a minimal group devoid of a history of intergroup relationships. The finding that only intragroup cooperation but not intergroup aggression emerged in the minimal group experiments strongly suggests that intergroup aggression involves a psychological mechanism that is independent from that of intragroup cooperation. We further discuss the implications of these findings on real-world politics and military strategy.

## Introduction

According to some archeological evidence (Wendorf, 1968), intergroup conflicts, of which the extreme form is warfare, have been observed in human societies since the late Paleolithic period (Keeley, 1996; Bowles, 2009; Pinker, 2011). Given the significance of intergroup conflicts and aggression in human societies, studies in various social science disciplines have sought causal mechanisms of intergroup conflicts (Waltz, 1959; Schelling, 1980). In social psychology in particular, intergroup conflicts are considered to be based on the psychological mechanism of intergroup bias (Brewer, 1979). This social psychology account of intergroup conflicts has recently been complemented by formal models of evolution (e.g., Choi and Bowles, 2007).

Social psychology research has shown that people have a tendency to cooperate with ingroup members and aggress against outgroup members; this phenomenon is called the intergroup bias (Hewstone et al., 2002). It has been argued this intergroup bias is omnipresent and observed even in a social vacuum called the minimal group situation (Tajfel et al., 1971), which is devoid of any form of conflict over tangible resources. Despite the absence of instrumental reasons, a large number of studies reported that intergroup bias occurs even in minimal groups (Brewer, 1979; Diehl, 1990). These results have been replicated across cultures (Yamagishi et al., 2008; Falk et al., 2014). Furthermore, the same pattern has been observed in students (Lemyre and Smith, 1985), young children (Fehr et al., 2008), and non-student samples (Mifune and Yamagishi, 2015).

The universal nature of intergroup bias observed in social psychology experiments has provided, at least partly, an impetus for evolutionary minded theorists to build mathematical models of the evolution of intergroup bias, including both intragroup cooperation and intergroup aggression. The evolution of cooperation with individuals beyond kin or partners in reciprocal relationships is hard to explain with the traditional kin-selection model or the reciprocal model of altruism (Hamilton, 1964; Trivers, 1971), and yet, it is frequently observed in humans (Fehr and Fischbacher, 2003). Thus, large-scale cooperation with strangers is one of the biggest puzzles in human evolution (Pennisi, 2005). Currently, this puzzle has been addressed in two theoretical lines. The first theoretical approach seeks the evolutionary foundation of cooperation beyond kin and reciprocal relationships in the personal benefits the cooperators themselves obtain through establishment of reputation and resulting positive behavior from other individuals in general (Nowak and Sigmund, 1998; Ohtsuki and Iwasa, 2006). The general exchange model of intergroup bias by Yamagishi et al. (1999), Yamagishi and Mifune (2008), and Mifune et al. (2010) in social psychology is based on this indirect reciprocity model of the evolution of cooperation.

The second theoretical approach in evolutionary modeling does not attribute the evolution of cooperation beyond kin and reciprocal relationships to its resultant personal gains. Rather, it explains the evolution of large-scale cooperation in terms of the group-level rather than individual-level fitness maximization. The model that provides an evolutionary foundation to the psychological interpretation of the intergroup bias is a form of the group-selection model called the parochial altruism (PA) model of the co-evolution of intragroup cooperation and intergroup aggression. According to Price’s equation (Price, 1970, 1972), it is argued that costly cooperation toward ingroup members could evolve if the intergroup variability in fitness (group-level selection pressure) is larger than the intragroup variability (individual-level selection pressure). Although this is not the case for almost all other organisms, some theorists argue that group-level selection pressures could exceed individual-level ones, especially in the case of humans (Wilson and Sober, 1994; Sober and Wilson, 1998; Gintis, 2000; Boyd et al., 2003; Henrich, 2004). The within-group variance would be reduced through the evolution of conformity (Henrich and Boyd, 1998; McElreath et al., 2003). Furthermore, intergroup aggression (e.g., warfare) enhances the fitness variability between groups (Henrich, 2004). The PA model further adds the logic of the co-evolution of intragroup cooperation and intergroup aggression, such that the fitness advantage of intragroup cooperation is enhanced by the presence of intergroup aggression (Choi and Bowles, 2007). A simulation study of the PA model lends support to the argument that cooperation toward ingroup members could only evolve when aggression toward outgroup members is present (Choi and Bowles, 2007). A psychological implication of this model is that humans have a built-in inclination toward cooperation with ingroup members together with aggression directed to outgroup members. Furthermore, this inclination is argued to be triggered by cues of intergroup relations, no matter if the cues involve actually existing groups (Bernhard et al., 2006; Reimers and Diekhof, 2015) or in nominal and arbitrarily created groups (Abbink et al., 2012; Baumgartner et al., 2012). This logic leads us to the prediction of outgroup-directed aggression, even in minimal groups.

A psychological implication of the above PA model account of intergroup bias, particularly intergroup aggression, is that it is based on the motivation of spite toward outgroup members. That is, intergroup aggression is a means by which to maximize the relative fitness difference between groups. The group-selection model of evolution argues that outgroup-directed aggression can evolve when it increases the relative gains of the ingroup compared to the outgroup, and the PA model adds that intergroup aggression enhances the fitness advantage of ingroup cooperation, and thus resulted in intergroup aggression and ingroup cooperation evolving hand-in-hand. Spiteful preference or competitive social value orientation (Messick and McClintock, 1968; Van Lange et al., 1997) toward the outgroup is considered a psychological means to make individuals engage in this evolutionary adaptive behavior. Despite this theoretical prediction, the evidence against it is mounting as shown in a meta-analysis conducted by Balliet et al. (2014). For example, studies using the allocation of a non-monetary negative resource (e.g., unpleasant noise), revealed that intergroup bias rarely occurred in minimal groups; this standard finding is often called the positive-negative asymmetry effect (Mummendey and Otten, 1998; Buhl, 1999). Another example comes from the intergroup prisoner’s dilemma-maximizing difference (IPD-MD) game (Halevy et al., 2008). In the IPD-MD game participants are able to choose between maximizing their own gains, improving ingroup benefits (i.e., ingroup cooperation only), and improving ingroup benefits while reducing outgroup benefits at the same time (i.e., both ingroup cooperation and outgroup aggression). Although some studies show that the third choice of ingroup cooperation and outgroup aggression is promoted under cognitive load (De Dreu et al., 2015) or in a non-retaliation situation (Cacault et al., 2015), the majority of studies using IPD-MD indicate that the overwhelming majority of participants choose the option of pure ingroup cooperation while rarely choosing the option of simultaneous ingroup cooperation and outgroup aggression (Halevy et al., 2008, 2012; De Dreu, 2010; De Dreu et al., 2010). Thus, at least up to now, spite-based outgroup aggression is rarely observed in laboratory experiments (Yamagishi and Mifune, 2016).

Despite the paucity of evidence of unchallenged, non-instrumental, spite-based intergroup aggression predicted by the PA model, intergroup conflicts are ubiquitous in our social life. This contrast between experimental evidence and real world conflicts suggests that the intergroup conflicts plaguing our social world are not based on the evolutionary-based human psychology predicted by the PA model. Rather, they are more likely consequences of real intergroup conflicts over tangible resources, moral righteousness, or a history of continuous fighting between groups. The absence of spite-based intergroup aggression in minimal groups in which either conflict over tangible resources, moral differences, or history of fighting is absent, strongly suggests that humans are not likely to be endowed with an evolutionary-based inclination toward intergroup aggression *per se*. It is likely that humans who continuously spend their social life in groups that are often in conflict with other groups have developed a fear-induced belief that they need to defend themselves from potential aggression from other groups, and this belief often prompts group members to engage in aggression toward potential aggressors in self-defense. In fact, Simunovic et al. (2013) compared the frequency of spite-based aggression and fear-based aggression and demonstrated that the overwhelming majority of aggressive incidents in the minimal group situation were fear-based rather than spite-based. Furthermore, research on the interindividual-intergroup discontinuity has shown that people have a naïve belief that groups are more competitive than individuals (Pemberton et al., 1996), and hence exhibit defensive non-cooperation in intergroup rather than inter-individual interactions in the prisoner’s dilemma situation (Wildschut et al., 2003; Wildschut and Insko, 2007). Although most people in modern times, living in democratic societies, do not actively or spitefully attack outgroup members (Yamagishi and Mifune, 2016), it is possible that they experience fear toward outgroups because of the intensity of historical intergroup conflicts. In this study, we focused on fear-based intergroup aggression and investigated if experimental participants exhibit aggression toward members of a minimal outgroup compared to a minimal ingroup.

For the purpose of studying fear-based aggression, we used the PSG developed by Simunovic et al. (2013). In the PSG, participants are paired with another participant, and each is given an initial monetary endowment (e.g., JPY 1,500 in Simunovic et al., 2013). Each participant then decides whether or not to push a button on a PC screen within a certain timeframe (e.g., 60 s) without any feedback about the other participant’s behavior. Both participants receive the initial endowment as is if they both restrain from pushing the button for the time duration. When one or both of the participants pushes the button during the time duration, the one who pushes the button (or, the one who does so first, if both participants push the button) inflicts a large cost on the other participant (e.g., JPY 1,000) by paying a smaller cost (e.g., JPY 100). These costs are subtracted from the initial endowment. Furthermore, pushing the button first preempts the other participant from retaliating. That is, the participant who faces an opponent who has pushed the button faster is deprived of the capability of successfully pushing the button. The participant who pushed the button faster nullifies the actions of their opponent—the second participant suffers a larger cost, and furthermore, cannot inflict the same larger cost upon the first. That is, the first attack eliminates the attacked party’s capability to retaliate. In this PSG, participants motivated by spite should attack their opponent because the relative payoff of a successful attack (i.e., the difference between 1,400 yen and 500 yen) is larger than that of mutual non-attack (1,500 yen, 1,500 yen). Fear also operates as a motivation to attack in this game. Participants who expect an attack from their opponent would try to push the button before it is too late to defend themselves from a potential attack from their opponent.

While logically either spite or fear motivate the participants’ attack (button pushing) in the PSG, Simunovic et al.’s (2013) findings indicate that fear is the dominant motivation for attack. They compared the standard PSG (explained above) with a unilateral condition of the game in which only one participant was allowed to push the button. In the unilateral condition, participants’ expectation of being attacked (i.e., fear) was removed from the experimental condition, leaving only spite as the motivation for attack. Results showed that almost half of the participants pushed the button in the standard condition while only one participant (out of 26) pushed the button in the unilateral condition. Furthermore, Simunovic et al. (2013) used the standard version of the game to compare the attack rate toward an ingroup member and an outgroup member with minimal groups. Results showed that the attack rate toward outgroup members (32%) did not significantly differ from that of ingroup members (29%).

While Simunovic et al. (2013) findings are impressive, theirs is the only study that utilized the PSG to study fear-based aggression as compared to spite-based aggression, and thus the robustness of the findings have not been fully established. The primary objective of this study is to demonstrate that the pattern found by Simunovic et al. (2013) can be replicated with a different set of parameters. For this purpose, we used different values for endowment (JPY 1,000 instead of 1,500) and damage imposed on the attacked party (JPY 500 instead of 1,000). This parameter change may weaken fear, and therefore the attack rate may decrease. It cannot be predicted, however, whether lowered fear causes an overall decrease in attack rate, a decrease in attack rate specific to the outgroup partner, or both. If we succeed in replicating the findings—(a) practical non-existence of spite-based aggression, (b) substantial fear-based aggression, and (c) the absence of differences in the attack rate toward the outgroup and the ingroup—with this set of parameters, we will be better assured that the finding is not limited to situations where fear of attack is particularly strong. With this assurance, we will finally speculate on the possible implications of the findings for intergroup conflict in general and for the co-evolution of intragroup cooperation and intergroup aggression in particular.

## Materials and Methods

This study was reviewed and approved by the ethical committee at Hokkaido University Center for Experimental Research in Social Sciences; in accordance with the Declaration of Helsinki, all participants provided written informed consent.

### Participants and Procedure

We first conducted a power analysis and found that 160 participants would be sufficient to correctly detect a substantial ingroup–outgroup difference in the attack rate of 20 percentage point at α = 0.05 with power above 0.8. Actually, 176 undergraduate students (100 males and 76 females) at Hokkaido University in Japan were randomly recruited from a large participant pool; women constituted 42% of participants in the outgroup-partner and 44% in the ingroup-partner condition (χ^2^(1) = 0.09, *p* = 0.761]. Cash rewards were emphasized as incentives for their participation. Monetary incentives were used to recruit the participants, while no course credit was offered.

Four to eight participants took part in a session. Upon arrival at the laboratory complex, they were provided with an ID number and led into isolated compartments one at a time and sat in front of a computer. Their only contact during the experiment was with an experimental assistant, who did not know the participants’ identification numbers and thus would not be able to match any of the participants to their behavior in the game. By assuring their anonymity this way, we sought to diminish participants’ responses based on demand characteristics and/or self-presentation, which are known to promote aggressive behavior in laboratory experiments (Tedeschi and Quigley, 1996; Ritter and Eslea, 2005).

The first task participants performed in the experiment was a “picture preference test.” Twenty-eight pairs of paintings, one by Wassily Kandinsky, and the other by Paul Klee, were consecutively displayed on the participants’ computer screens. The participants then decided, on each pair, which painting they liked more. They were then allotted into either the Klee or the Kandinsky group. We asked the participants about their attitudes toward ingroup and outgroup members, using a minimal group identity scale (adapted to the minimal group situation by Yamagishi and Mifune, 2008, based on Grieve and Hogg, 1999), consisting of items such as: “How much do you like Klee- (or Kandinsky-) group members?” “In terms of your general beliefs and attitudes, how similar do you feel you are to other Klee- (or Kandinsky-) group members in general?”

### Preemptive Strike Game

Once all the participants had been assigned group memberships, they were given instructions for the PSG. The instructions were framed in terms of dyadic decision-making concerning monetary outcomes, without reference to meaning-laden words such as “defense,” “opponent,” or “attack.”

In the instructions, it was explained that all participants had been given JPY 1,000 (∼USD 9 at the time of the experiment) by the experimenter. They would then be paired with another participant in another compartment, and would decide whether to push a button on their computer screens or not. They had a 30 s period during which they could enact their decisions. If both participants refrained from pushing the button, each participant would receive the initial endowment of JPY 1,000. The participant who pushed the button first paid JPY 100, while the other participant, regardless of whether they themselves pushed the button or not, incurred a cost of JPY 500 (half of the endowment size). It was made clear to the participants that only the action of the one who pushed the button would count.

To examine if participants had a preference for either an ingroup member or an outgroup member for playing the PSG with, we asked 141 of the 176 participants, before they were matched with a partner, if they would prefer an ingroup member or an outgroup member as their game partner, or if they had no preference at all. We manipulated group membership as a between-participants factor. Once all the participants in a session finished reading the instructions, they were matched with either an ingroup member or an outgroup member, and played one round of the computer-mediated PSG with the matched partner. The assignment of the partner’s group membership was made randomly even when participants were asked about their partner preference. The participants understood that the real assignment would be made randomly rather than based on their partner preference. Each participant could push the button during the first 10 s regardless of whether the partner had pushed the button or not, although the button push did not have any effect when the partner had already pushed the button. This design feature allowed us to examine the participants’ willingness to attack, independently of how fast they could move. According to Simunovic et al. (2013), almost all participants who pushed the button did so within the first second of the game, and thus we could capture the intentions of the relatively slow responders to attack with this design. We measured reaction time to the third decimal place to compare matched participants’ attack effectiveness. Feedback about the outcome of the game was given to the participants either at the end of the 30 s (for mutual non-attack), at the end of the 10 s period (in case of unilateral attack), or immediately after the slower participant’s button pushing (in case of mutual attack). They were informed about the outcome of the game over the computer screen. After the game, participants provided answers to a post-experimental questionnaire, received their payments, and were led out of the laboratory one at a time.

## Results

All raw data used in the analysis is in **Supplementary Material**.

### Group Identity

We calculated the relative identity score defined by the mean identity score toward the ingroup minus that of the outgroup. The mean relative identity score was 0.31 (*SD* = 1.03), which was significantly greater than zero [*t*(175) = 4.04, *p* < 0.0001, Cohen’s *d* = 0.25]. There were no main or interaction effects involving gender (*p*s > 0.15). This result shows that the minimal group manipulation (i.e., identification with the ingroup) was successful.

### Attack Rate

**FIGURE 1:**
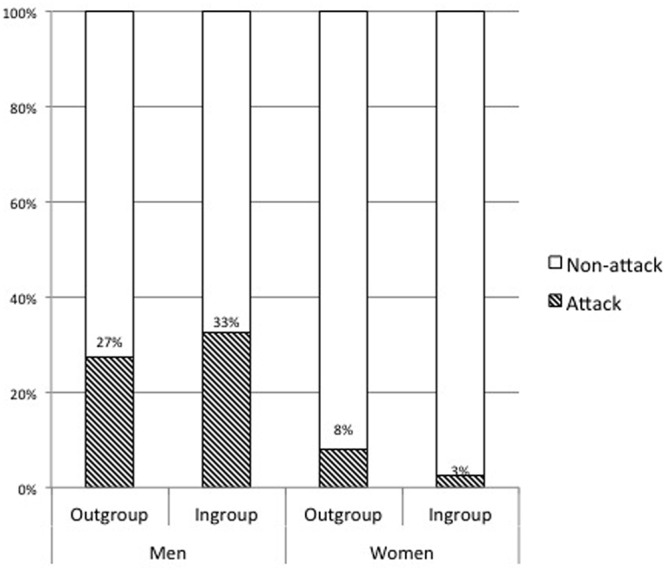
**The attack rate toward ingroup and outgroup members**.

The total percentage of attack (button pushing) was 19.3% (95% CI [13.4, 25.2]). This attack rate did not vary between group memberships: as in the previous study, there was no difference in the attack rate when the participants were paired with an ingroup (*N* = 88, 19.3%) or an outgroup member [*N* = 88, 19.3%, χ^2^(1) = 0, *p* = 1, Cramer’s *V* = 0]. The use of categorical independent variables and the lack of correlation between the two independent variables (partner group and gender) meant the data met the assumptions of logistic regression analysis (no multicollinearity, a linear relationship between independent and dependent odds ratio) in addition to the assumptions of a large and independent sample. We performed a logistic regression analysis of attack by the partner group and gender of the participant. The main effect of gender was significant (Wald *χ*^2^ = 4.60, *p* = 0.032, adjusted *R*^2^ = 0.18). Men (30%) were more likely to attack than women (5.26%). The main effect of the partner group or the interaction effect was not significant (*p*s > 0.26) (see **Figure 1**).

### Partner Preference

More than two–thirds (70.63%) of the participants who responded to the partner preference question (*N* = 143) stated that they did not care about the group membership of the partner. About a quarter (23.78%) preferred to play with an ingroup member and only eight (5.59%) preferred to play with an outgroup member. Because the number of participants who preferred an outgroup member was small, making the use of logistic regression with interactions problematic, we combined the no-preference and outgroup-preference to form a combined not-ingroup preference category. No gender difference was found in partner preference [*χ*^2^(1, *N* = 143) = 0.055, *p* = 0.815]. In the logistic regression analysis of attack using partner group, partner preference, gender, and their interactions as independent variables, none of the interaction effects were significant (*p* > 0.43). We thus dropped all the interactions from the logistic regression analysis, and found that only gender was significant (Wald *χ*^2^ = 12.49, *p* < 0.001); neither the main effect of partner preference nor partner group was significant (*p* > 0.59).

### Post-experimental Questions

In a post-experimental questionnaire, we asked participants how strongly they were concerned, before the game actually started, about the possibility that the partner would push the button faster. The mean response to this question on a 7-point scale was 5.47 (*SD* = 1.89) among those who pushed the button and 3.72 (*SD* = 1.61) among those who did not. The difference was highly significant [*t*(174) = 5.48, *p* < 0.0001, Cohen’s *d* = 1.04]. There were no main or interaction effects of gender (*p*s > 0.83) in an ANOVA adding gender and partner-group by gender interaction as independent variables. Furthermore, a similar question about how anxious they were about the possibility of the partner pushing the button faster received similar responses [*M*_attack_ = 4.62, *SD* = 1.89; *M*_non attack_ = 3.80, *SD* = 1.62; *t*(174) = 2.57, *p* = 0.011, Cohen’s *d* = 0.49]. Again, there were also no main or interaction effects involving gender (*p*s > 0.28). These results provide support for the fear-based nature of the preemptive attack in the PSG. We also asked the participants how satisfied they would be if both participants refrained from pushing the button and earned JPY 1,000 each, and how satisfied they would be if they pushed the button first and earned JPY 900. The participants’ satisfaction with the latter outcome should be higher than that of the former if they were motivated by spite. The result was completely opposite to this PA model-based prediction (see **Table 1**). Only 5 of 176 participants who responded to these questions expressed greater satisfaction with the successful attack outcome than the mutual restraints outcome, and 157 of 176 expressed greater satisfaction with the latter outcome compared to the former. The remaining 14 participants expressed the same level of satisfaction. Half of the participants who pushed the button (17 of 34) chose that option despite their higher satisfaction with the mutual restraints outcome. Twelve of those who pushed the button expressed the same level of satisfaction with the two outcomes, and only five of them responded consistently with the PA model prediction, expressing a higher satisfaction with the successful attack outcome than the mutual constraints outcome and pushing the button. The overall picture emerging from these post-experimental questionnaire analyses is that only very few participants (5 of 176; 3 of 88 who played with the outgroup, and 2 of 88 who played with the ingroup) engaged in a preemptive strike out of spite, and the majority of them did so out of fear-based self-defense.

**Table 1:** The number of participants answering the satisfaction question.

	Successful attack > Mutual restraints	Indifferent	Successful attack < Mutual restraints
All data (*N* = 176)	5 (2.84%)	14 (7.95%)	157 (89.2%)
Male (*N* = 100)	5 (5%)	11 (11%)	84 (84%)
Female (*N* = 76)	0 (0%)	3 (3.95%)	73 (96.05%)
Attacker (*N* = 34)	5 (14.71%)	12 (35.29%)	17 (50%)
Male attacker (*N* = 30)	5 (16.67%)	11 (36.67%)	14 (46.67%)
Female attacker (*N* = 4)	0 (0%)	1 (25%)	3 (75%)

## Discussion

The current study largely replicated the results of Simunovic et al. (2013). First, a significant portion of the participants pushed the attack button. A noticeable difference from the previous study is that the overall attack rate (i.e., 19%, 95% CI [13.4, 25.2]) in the current study was smaller than that of the previous study [50% in Study 1, *χ*^2^(1) = 13.96, *p* < 0.001, and 30% in Study 2, *χ*^2^(1) = 4.97, *p* < 0.05 (Simunovic et al., 2013), both of which significantly differ from the current attack ratio of 19% in the current study]. This difference may reflect the fact that the cost of being attacked and thus the motivation of defensive aggression were smaller in the current study (JPY 500, or half of the endowment in the current study versus JPY 1,000 or two-thirds of the endowment in the previous study). This difference suggests that the greater the suffering from an attack from the opponent the more willing one is to defend their self-interest by preemptively eliminating the potential of an attack from the opponent (cf., Mifune et al., 2016). This possibility is worthy of further investigation in another study in which the damage caused by an attack from the opponent is manipulated in a single experiment.

Simunovic et al. (2013) did not report a gender difference in the attack rate because they did not find one [Study 1, *M* = 63.2% and *F* = 30.8%, *χ*^2^(1, *N* = 32) = 3.24, *p* = 0.076; Study 2, *M* = 31.8% and *F* = 28.8%. *χ*^2^(1, *N* = 132) = 0.14, *p* = 0.705]. Knowing this, we did not expect any gender difference in this study. Thus, the big gender difference we found in this study was a surprise. Despite the strong main effect, however, none of the interaction effects involving gender were significant, and thus our conclusions need not be altered depending on the participant’s gender. Below we offer some *post hoc* speculations on how gender made a difference in this study, but not in the previous study (Simunovic et al., 2013). Participants were recruited from the same participant pool on the promise of monetary compensation. The gender constitution was about the same in both studies [43.2% female in the current study and 50% in the previous study; *χ*^2^ (1, *N* = 308) = 1.41, *p* = 0.235]. These sample compositions therefore provide no explanations for the difference. The major difference between the two studies is in the cost of being preemptively attacked; it was much smaller in this study than in the previous study. The resulting smaller fear of preemptive attack in this study is thus the primary candidate for the gender difference in this study that did not exist in the previous study. It is possible that women are more sensitive to fear of exploitation (Simpson and Van Vugt, 2009) and to the moral principle of no harm (Graham et al., 2011) than men, and the defensive attack was a result of the balance between the two. The smaller cost of exploitation in the current study might have meant less motivation for defensive aggression and a stronger moral principle against harm for trivial reasons especially for women who are more sensitive to them than men, resulting in weaker fear-based defensive aggression than men. An alternative explanation could be men’s overconfidence. Johnson et al. (2006) found that overconfidence in one’s own relative standing was a significant contributor to attack rate in an experimental wargame. Furthermore, this trend was more pronounced in males than in females, who mostly evaluated themselves as average. In our game, this could have meant male participants felt they would push the button more quickly than any opponent, while female participants did not. No matter if any of these possibilities are right or wrong, gender differences in defensive aggression is an important topic, given the general importance of gender differences and a small but persistent tendency for males to exhibit more aggression across situations (Eagly and Steffen, 1986; Eagly and Wood, 1991; Baron and Richardson, 1994). This gender difference is also known to be stronger in the group context (Goodall, 1986; Chagnon, 1988; De Waal, 2005), and the male warrior hypothesis (Van Vugt et al., 2007; Van Vugt, 2009) may play some role in this endeavor, although the motivation of spite that is the central force in the male warrior hypothesis does not seem to play a big role in the PSG.

Despite this puzzling gender difference, we succeeded in replicating the previous finding of no intergroup bias in defensive aggression. The attack rate toward outgroup members was not significantly different from that toward ingroup members. With this in mind, let us speculate on their implications for the evolution of intra-group cooperation and inter-group aggression. First, intra-group cooperation is omnipresent both in the laboratory and the real world. It occurs even in the minimal group situation. This suggests that the human inclination to cooperate with ingroup members is deeply rooted, and is effectively activated with a relatively minor cue of groupness. On the other hand, intergroup aggression is hardly observed in laboratory experiments, especially when nominal, rather than real groups are involved, despite the fact that it is often observed in social life sometimes with serious consequences. In other words, inter-group aggression is not likely internally activated by cue-driven psychological mechanisms. Rather, it requires socially and historically embedded groups in which a history of past atrocities is shared by group members. These implications cast a serious doubt on the validity of the PA model of intra-group cooperation and inter-group aggression. Given the differences between intra-group cooperation and inter-group aggression, it is more natural to think that the two involve rather mutually independent psychological mechanisms; intra-group cooperation involving evolved neuropsychological mechanisms, while inter-group aggression involving beliefs about the history of intergroup relations and the expectations of the behavior of the outgroup. This conclusion, however, does not exclude the possibility that such belief formation is facilitated by the evolutionary-based group psychology.

Before closing, we would like to discuss limitations of our study. The participants of our study are semi-WEIRD; although not Western, they are members of an educated, industrialized, rich, and democratic society in which norms against the use of aggression prevail. Another study using a design that reduces the participants’ concerns with the use of aggression would definitely help broaden the scope of the conclusions. Another possible limitation is the lack of face-to-face communication among the same-group participants, because group members often conspire to make group-based decisions such as preemptive strikes (cf., Mifune et al., 2016). Aggression may be against norms when performed by an individual, but it can be condoned or even praised as the norm when it is agreed upon by group members. Combining face-to-face communication with the presence of a decision-making leader who can mobilize the members’ fear to justify an otherwise unjustifiable preemptive strike would be an exciting topic for future study.

Traditionally, studies of inter-group aggression in social psychology have been dominated by designs that force intra-group cooperation and inter-group aggression together, as inseparable sides of the same coin. For example, it was impossible for the resources provided to the ingroup to vary independently of the resources provided to the outgroup in the resource allocation paradigm originating in the seminal work by Tajfel et al. (1971). In studies such as the intra- and inter-group PSG, in which this design constraint was removed, participants often cooperated with an ingroup member at higher levels than with an outgroup member or a partner whose group membership was not known. However, the cooperation level with an outgroup member was never found to be below the level they cooperated with an unknown group member (Yamagishi and Mifune, 2016). Furthermore, participants in most IPD-MD studies chose the option that provided resources to the ingroup at a cost to themselves but rarely chose another option that reduced resources of the outgroup while providing resources to the ingroup. Given the findings of the current and the previous studies indicating independence of inter-group aggression and intra-group cooperation, future studies will benefit by shifting their focus from the motivation of spite to the motivation of fear. The lack of inter-group bias in fear in the minimal group situation that was confirmed in this study provides a benchmark for examining the effects of other factors in producing fear of aggression from other groups. The comparison of the attack rates in the current study and in Simunovic et al.’s (2013) study suggests one such factor, that is, the seriousness of the damage caused by the opponent’s aggression. Another interesting and potentially important topic for future study concerns the possibility that unilateral disarmament reduces fear-based aggression from the partner, as shown in the almost complete lack of aggression in the unilateral aggression condition. This issue is at the core of the current public dispute in Japan on the abolition of Article 9 of her constitution that prohibits Japan’s capability to use military power as a means of international conflict resolution. Supporters of Article 9 share the belief that it will promote peace; while those who want to abolish it believe that it will more likely promote war because it enhances the temptation for potential enemies to intimidate Japan. If used in a proper manner, the PSG will provide useful tools to provide scientific answers to this issue and similar questions of vital interest to politicians, military strategists, psychologists, and ordinary citizens.

## Author Contributions

NM, DS, and TY conceived the design, and developed the instruments. NM and DS conducted the experiment. NM, DS, and TY analyzed the data and wrote the whole part of manuscript.

## Conflict of Interest Statement

The authors declare that the research was conducted in the absence of any commercial or financial relationships that could be construed as a potential conflict of interest.

## Supplementary Material

SUPPLEMENTARY DATA SHEET 1 **|Raw data**.

## Abbreviations

PA model: parochial altruism model
IPD-MD: intergroup prisoner’s dilemma maximizing difference
PSG: preemptive strike game
